# Evaluating the Potential Efficacy of Invasive Lionfish (*Pterois volitans*) Removals

**DOI:** 10.1371/journal.pone.0019666

**Published:** 2011-05-10

**Authors:** Andrew B. Barbour, Michael S. Allen, Thomas K. Frazer, Krista D. Sherman

**Affiliations:** 1 Fisheries and Aquatic Sciences Program, School of Forest Resources and Conservation, The University of Florida, Gainesville, Florida, United States of America; 2 Parks and Science, The Bahamas National Trust, Nassau, The Bahamas; Institute of Marine Research, Norway

## Abstract

The lionfish, *Pterois volitans* (Linnaeus) and *Pterois miles* (Bennett), invasion of the Western Atlantic Ocean, Caribbean Sea and Gulf of Mexico has the potential to alter aquatic communities and represents a legitimate ecological concern. Several local removal programs have been initiated to control this invasion, but it is not known whether removal efforts can substantially reduce lionfish numbers to ameliorate these concerns. We used an age-structured population model to evaluate the potential efficacy of lionfish removal programs and identified critical data gaps for future studies. We used high and low estimates for uncertain parameters including: length at 50% vulnerability to harvest (*L_vul_*), instantaneous natural mortality (*M*), and the Goodyear compensation ratio (*CR*). The model predicted an annual exploitation rate between 35 and 65% would be required to cause recruitment overfishing on lionfish populations for our baseline parameter estimates for *M* and *CR* (0.5 and 15). Lionfish quickly recovered from high removal rates, reaching 90% of unfished biomass six years after a 50-year simulated removal program. Quantifying lionfish natural mortality and the size-selective vulnerability to harvest are the most important knowledge gaps for future research. We suggest complete eradication of lionfish through fishing is unlikely, and substantial reduction of adult abundance will require a long-term commitment and may be feasible only in small, localized areas where annual exploitation can be intense over multiple consecutive years.

## Introduction

Invasive Indo-Pacific lionfishes, *Pterois volitans* (Linnaeus) and *Pterois miles* (Bennett), are established in the offshore waters of the Southeast United States, Caribbean, and are presently invading the Gulf of Mexico and South America [1], [2], [3], [4]. The lionfish invasion is concerning due to the danger of human health risks by venomous lionfish spines and because of numerous potential ecological effects on native hard-bottom, mangrove, seagrass, and coral reef communities. For example, lionfish have been shown to reduce native fish recruitment on experimental patch reefs in The Bahamas [5] and reductions in reef fish recruitment may be exacerbated by lionfish predation upon juvenile native fish in important nursery habitats such as mangroves and seagrass beds [6] possibly limiting the supply of economically important reef fish recruits [7].

Through these mechanisms lionfish may be contributing to widespread regime-shifts on Caribbean coral reefs by consuming herbivores responsible for controlling macroalgal production [5], [6], [8]. The effects of the lionfish invasion will likely continue to spread, as lionfish have extensive dispersal capabilities [7], [9] and are thought only to be limited in range by temperatures below 10.0°C [10]. Furthermore, known instances of predation upon lionfish in the Western Atlantic are rare and limited to incidental natural occurrences of predation by such species as groupers (Serranidae) [11] and green moray eels, *Gymnothorax funebris* (Ranzani) (KD Sherman, pers. obs.). Moreover, predation on juvenile lionfish by common reef predatory species in laboratory trials suggests low predation vulnerability [12]. This suggests lionfish populations lack a top-down control mechanism to regulate their population size in their invasive range.

As a result of this established and destructive invasion, many countries have instituted lionfish removal programs. These programs include initiatives such as creating a special license to allow the spearing of lionfish on nearshore reefs and lionfish kill orders intended to involve the general public in removal efforts [13]. The largest initiatives involve using recreational divers to remove lionfish during derby events, and focusing commercial divers and fishers on harvesting lionfish as a food fish [7]. Developing lionfish as a commercial or recreational fishery has been proposed as a potential long-term solution [7], but it is not yet fully understood what level of exploitation might be required to control lionfish populations.

To date, only one evaluation has explored the level of harvest required to substantially reduce lionfish population size. This study [14] utilized a stage-based matrix lionfish population model and indicated that decreasing lionfish abundance would require monthly removals of 27% of the adult lionfish population. The study also reported that this required adult exploitation rate could be significantly reduced if juveniles were removed from the population [14]. Their model was density-independent, which is appropriate for a recently introduced lionfish population. Thus, the model assumed no compensation in recruitment after fishing or after the particular population reached a level where density-dependence would occur, as would be expected for a maturing and well-established invasive species population. Lionfish have now been in the Atlantic basin for over 10 years and have reached high densities (>450 fish per hectare) in some locations [7], [15], however, population assessments of abundance are generally lacking. Nevertheless lionfish recruitment per adult would be expected to increase as adult abundance is reduced by removal efforts via recruitment compensation as is typical of established fish populations [e.g. 16]. Thus, it is essential that removal practices and policies be evaluated for scenarios where recruitment compensation occurs.

In this study we used an age-structured population model to evaluate the potential for removal programs to overfish lionfish populations, while identifying key data gaps to guide future research. Existing lionfish life history information was compiled to develop the model, and various harvest rates were applied to evaluate the efficacy of harvest as a top-down control mechanism. Harvest rates were evaluated for upper and lower estimates of uncertain and unknown parameters. The results of the model can be used to inform the best possible management strategies under current knowledge while guiding future work to reduce key uncertainties.

## Methods

### Model Description

Only small numbers of *Pterois miles* have been documented along the Southeast United States [17] with no captures to date in The Bahamas [18]. Lionfish are hereafter referred to as inclusive of both *P. miles* and *P. volitans*. For the purpose of this modeling exercise, life history parameters were derived primarily from *P. volitans*. We assumed that given the taxonomic similarity between *P. volitans* and *P. miles* (two closely related sympatric species), there would be no substantial life history differences [see 19] between the two species that might affect the overall outcome of this study.

The population model structure was identical to that published previously [20], [21], and it predicted equilibrium recruitment and age-specific abundance under a variety of harvest rates. Survival schedules incorporated natural and harvest mortalities. Harvest was driven by a stated exploitation rate and length-based vulnerability to removal efforts. Fecundity was expressed as a function of fish weight and the collective fecundity for a given year was reduced by all mortality sources. The model included ages 1–20 and was constructed in Excel®.

Equilibrium recruitment was calculated using a Botsford modification of a Beverton-Holt stock recruitment function [22], [23], [24] as described elsewhere [20]. This simple formulation predicts equilibrium recruitment as a function of the fishing mortality rate. The model predicted the equilibrium age-1 recruits (R*_eq_*) of an exploited population and is summarized as [20]

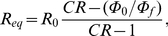
(1)where *R_0_* is the number of age-1 recruits of the unfished population at equilibrium, and *CR* is the Goodyear compensation ratio [25]. It is unknown if the current population is near the asymptotic unfished abundance, however, because lionfish populations have been established for over ten years in the Atlantic coastal waters, we initiated the simulation at unfished equilibrium. This is supported by simulation runs initialized at very low population size, which reached equilibrium recruitment in four to six years depending upon the value for *CR*. The *CR* is defined as the ratio of the recruits per spawner at very low population abundance relative to the recruits per spawner in the unfished equilibrium condition [25]. The parameter *R_0_* is the unfished age-1 recruitment at equilibrium and is simply a scaling parameter that does not influence model predictions.

The model used survivorship curves to calculate the survivors per recruit to each age. Survivorship to age *a* in the absence of fishing was found as
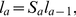
(2)where *S_a_* is the age-specific finite annual natural survival (i.e., *e^−M^*). Our survivorship schedules in the fished condition incorporated natural mortality and harvest as
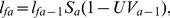
(3)where *l_fa_* is the survivorship in the fished condition, *U* is the finite annual exploitation rate, and *V_a_* is the age-specific vulnerability to harvest. We specified the proportion of fish vulnerable to harvest as
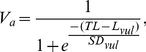
(4)where *TL* is the mean total length at age *a* as calculated from the von Bertalanffy growth model, *L_vul_* is the total length at 50% vulnerability to capture, and *SD_vul_* is the standard deviation of the logistic distribution for *L_vul_*. The term *V_a_* models increasing vulnerability with length, and *SD_vul_* specifies the steepness of the curve. Age-specific abundance (*N_a_*) was estimated as the product of the number of age-1 recruits (*R_eq_*) and the age-specific survivorship schedule.

Mean fish weight-at-age was used as an index of fecundity (egg production) as fecundity is directly proportional to weight-at-age. The age-specific fecundity (*f_a_*) was set to zero if weight-at-age was less than weight-at-maturity. To account for the cumulative effects of fishing on the reproductive capacity of the population, we used the incidence function for the unfished (*Φ_0_*) and fished (*Φ_f_*) egg production per recruit [20]. These incidence functions were calculated as
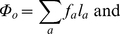
(5)

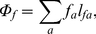
(6)where *f_a_* represents age-specific fecundity, and *l_a_* and *l_fa_* are the survivorship schedules of the unfished and fished states. We used the weighted spawning potential ratio (SPR) to evaluate the extent to which fishing mortality can reduce reproductive output of lionfish
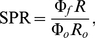
(7)where *Φ_0_* and *Φ_f_* are defined in Eqs. (5) and (6), and *R* is the recruitment at equilibrium in the fished condition. The weighted SPR measures the population for a given level of fishing mortality relative to the unfished condition, which is a commonly used reference to assess fisheries sustainability [26]. Recruitment overfishing is generally termed to occur when SPR is below about 0.4 [27]. For this study, we define recruitment overfishing as occurring when SPR≤0.35.

### Parameter Estimates

Parameter estimates used in the model simulation are shown in Table 1. Due to a lack of empirical data on *CR* for lionfish, we selected two values for *CR*, 5 and 15. These values represented biologically reasonable high and low estimates for species with similar life history traits (e.g., relatively short lived predators), based upon past meta-analyses [16], [28].

**Table 1:** Parameters used in the simulation model.

Parameter		Value	Data Source
Natural Mortality			
*M*	instantaneous adult natural mortality (yr^−1^)	0.2 and 0.5	Inferred
Fishing Mortality			
*U*	annual harvest exploitation rate	0.00 to 1.0	
Vulnerability			
*L_vul_*	length at 50% capture vulnerability (mm)	159 (age-1) and 259 (age-2)	Inferred
*SD_low_*	standard deviation of 50% capture vulnerability	10% of *L_vul_*	Inferred
Growth			
*L∞*	asymptotic length (mm)	425	This study
*K*	metabolic coefficient (yr^−1^)	0.47	This study
Length-Weight			
*a*	length-weight coefficient	2.89×10^−5^	This study
*b*	length-weight exponent	2.89	This study
Recruitment			
*R_o_*	average annual unfished recruitment	100	Scaling parameter
*CR*	Goodyear compensation ratio	5 and 15	Inferred
*W_mat_*	weight at maturity (kg)	0.07	Empirical Data

Age and growth data to determine values for *L_∞_* and *K* (Table 1) were obtained from lionfish collected in offshore waters of North Carolina by spearfishing, hand nets, hook and line, and trapping during 2004–2009 (data provided by J. Potts, NMFS [29]). Collection sites ranged between 27–45 m depth and were characterized as hard-bottom habitat comprised of rocky outcroppings. Sagittal otoliths were removed, dried, and embedded in epoxy. Otoliths were serially sectioned on a low-speed saw. The resulting sections were adhered to microscope slides and covered with a liquid cover-slip.

**Figure 1:**
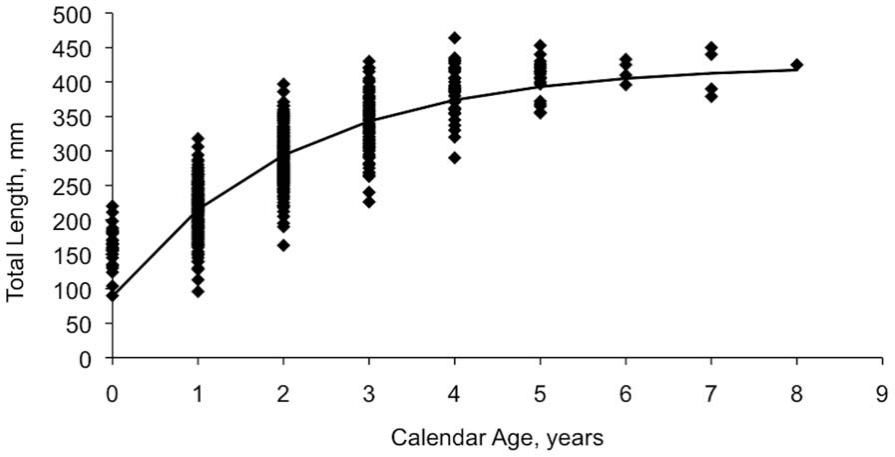
Length at age for lionfish collected from North Carolina. The von Bertalanffy growth curve is shown as calculated by the equation: 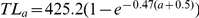.

Opaque zones were enumerated for each fish and width of the margin was noted. Opaque zone counts were converted to calendar ages based on timing of opaque zone completing and date of capture. A total of 774 fish were aged by a single person. Quality assurance was assured using a second “blind” reader to maintain >95% agreement. A von Bertalanffy growth curve was fitted using maximum likelihood estimation of the normal distribution (Figure 1), but we fixed *t_o_* at −0.5 because of likely overestimation of mean length at age-0 due to gear bias. The fitted value of *L_∞_* = 425 mm is consistent with data reported previously; for example, a past study [30] collected >1,000 lionfish with the largest fish measuring 424 mm TL; and Reef Environmental Education Foundation's (REEF) lionfish derbies, which have resulted in the collection of n = 2,349 lionfish, indicate that the largest fish measured 434 mm TL.

**Figure 2:**
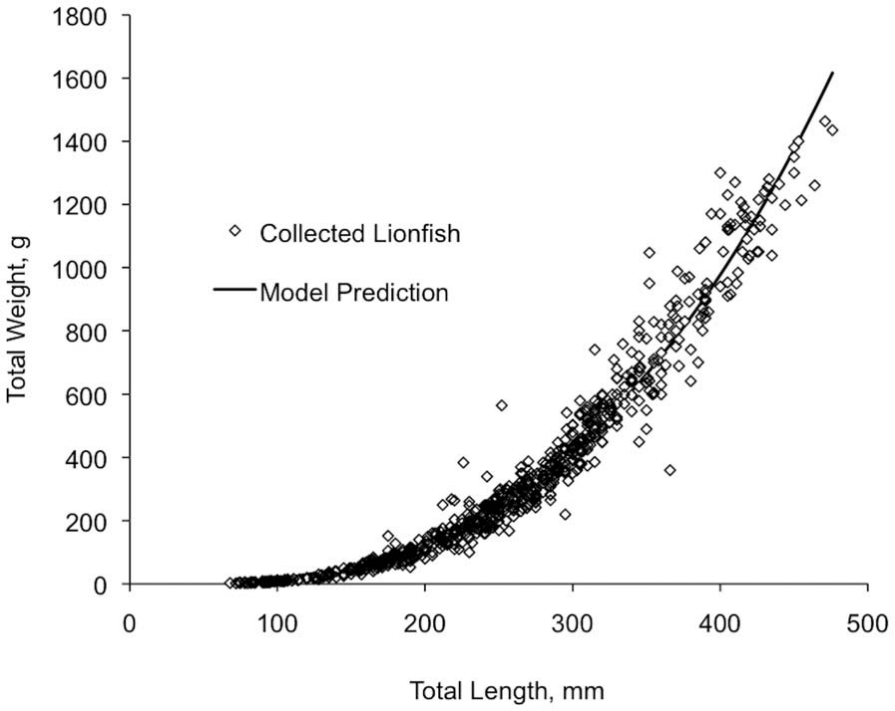
Lionfish length-weight relationship for lionfish collected from North Carolina. Lionfish total length (mm, x axis) and total weight (g, y axis) relationship and estimates of *a* (2.89×10^−5^) and *b* (2.89) growth parameters. Model predicted values calculated as: 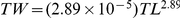.

To predict fish weight from length, *a* and *b* parameters were estimated from the dataset of 774 lionfish collected from the offshore waters of North Carolina. Using maximum likelihood of the normal distribution, values of 2.89×10^−5^ and 2.89 were estimated for the *a* and *b* growth parameters (Figure 2). Lionfish size at 50% maturity has been estimated at 100 mm TL for males (n = 927) and 175 mm TL for females (n = 718) through examination of gonadal tissue [12]. Age at 50% maturity was specified as age-1, which corresponded to a model-predicted total length of 159 mm and a weight at 50% maturity (*W_mat_*) of 0.07 kg (Table 1).

Instantaneous natural mortality (*M*) is unknown for lionfish. We used values of 0.2 and 0.5 for *M* (Table 1). A value of *M* = 0.5 is typical of short-lived fish and was similar to the value used in a past study [14]. The value of *M* = 0.2 is typical of longer-lived species and would be indicative of a fish with a 15–20 year life span: in captivity, the maximum lifespan of lionfish has been recorded as thirty years [29]. We included this range in *M* due to uncertainty in lionfish maximum age. Little data exist for lionfish in their native range, and they have not been present in the invaded range for enough time to allow estimation of maximum age, and thus natural mortality.

The total length at 50% vulnerability to removal efforts (*L_vul_*) has not been studied. We assumed that small fish would be less visible and less likely to be spotted during removal efforts than larger animals; a larger fish bias is typical of nearly all fishing gears. Lionfish are easily speared, but difficult to catch on hook-and-line. It is also difficult to spear small lionfish, meaning most capture of juveniles will require hand netting or other methods. Therefore, we evaluated harvest scenarios under two possible lengths at 50% vulnerability: 159 mm TL (age-1) and 259 mm TL (age-2, Table 1). Fish recruited to removal efforts according to the logistic function in the model (Eq. 4), and fully vulnerable fish were harvested at the rate of annual exploitation (*U*).

### Simulation Protocol

To evaluate the efficacy of lionfish harvest as a removal tool, we simulated a range of exploitation rates (*U* = 0.05–1.0 in 0.05 step increments). We applied these exploitation rates to a variety of scenarios in order to model the full range of uncertainties in model parameters. First, *L_vul_* was set at either 159 mm TL (age-1) or 259 mm TL (age-2). Then, values for *M* (0.2 or 0.5) and *CR* (5 or 15) were selected. All possible combinations were evaluated, and the equilibrium output metric was the annual finite exploitation rate (*U*) required to recruitment overfish the stock (*U_SPR<0.35_*). For each scenario we calculated the number of years to lionfish recovery following the removal of harvest, defined as a return to 90% of the unfished biomass. Calculating recovery time is important as many invasive removal programs are funded only for short periods of time. Additionally, to determine the sensitivity of SPR to uncertainty in model parameters, we modeled the effect of a 10% increase in individual parameter estimates on SPR.

## Results

The model indicated a high degree of variability in the annual finite exploitation rate (*U*) required to cause overfishing in lionfish populations. This variability depended on the uncertain parameter values of *L_vul_*, *M*, and *CR* (Table 2). Of the unknown parameters, *L_vul_* had the largest impact on exploitation rates that achieved overfishing (Table 3). Lower *L_vul_* values resulted in a substantially lower *U* being required for overfishing than higher values of *L_vul_* (Table 2). Thus, fishing gears that select small lionfish would be more effective at causing overfishing than those removing only large fish. Instantaneous natural mortality (*M*) had the second largest impact (Table 3), with a high *M* of 0.5 requiring considerably higher *U* than *M* = 0.2. This occurs because when natural mortality is low, exploitation strongly changes abundance. When natural mortality is high, fish naturally die at a high rate, so exploitation has a dampened effect on total abundance [20].

**Table 2:** Model results for all combinations of possible *L_vul_*, *M*, and *CR* parameter values.

*L_vul_* (mm)	M	CR	*U_SPR<0.35_*	Recovery (yrs) after *U_SPR_* Fishing
159	0.5	15	0.35	6
159	0.5	5	0.3	10
159	0.2	15	0.20	12
159	0.2	5	0.15	16
259	0.5	15	0.65	6
259	0.5	5	0.50	9
259	0.2	15	0.25	11
259	0.2	5	0.2	16

Model outputs include: (1) *U_SPR_*, defined as the finite annual exploitation rate (*U*) required to reduce SPR to or below 0.35; and (2) recovery (in years) after *U_SPR_*.

**Table 3:** Model sensitivity to increasing given parameters by 10%.

Parameter	SPR %Change
*M*	8%
*L_vul_*	11%
*SD_vul_*	0.0%
*CR*	1%
*K*	−6%
*a*	0%
*b*	−3%
*L_∞_*	−12%
*W_mat_*	0.0%

Sensitivity analysis performed with starting values of *L_vul_* = 159; *M* = 0.5; *CR* = 15; and *U* = 0.35 (Table 2).

There was considerable variability in years to recovery after the cessation of exploitation. Lionfish populations recovered fairly quickly (6–7 years) when *M* and recruitment compensation (*CR*) were high (0.5 and 15). Recovery took 15–25 years when lower parameter estimates (*M* = 0.2 and *CR* = 5) were used. If the higher parameter estimates represent realistic conditions for lionfish in the invaded range, high levels of sustained fishing mortality will be required to cause overfishing. Given that the oldest lionfish aged in this study was age-8, it is likely lionfish are short-lived predators and that our estimates of *M* = 0.5 and *CR* = 15 are appropriate. With these parameter values and an *L_vul_* of 159 mm SL, an annual exploitation of at least 35% would be required to cause recruitment overfishing. At an *L_vul_* of 259 mm SL, a 65% annual exploitation would be required to cause recruitment overfishing.

Results of the sensitivity analysis showed that uncertainty in *L_∞_*, *L_vul_*, *M*, and *K* had the greatest effect on SPR (Table 3). This study had estimates of *L_∞_* and *K* from age-growth information, but no data exist on *L_vul_* or *M*. The value of *L_vul_* is largely dependent on the type of removal efforts used. Spear guns are selective for larger lionfish (S. Green, pers. comm.), and a true fishery would be biased towards larger lionfish due to higher meat content. These size biases might be mitigated with targeted hand netting of small lionfish, as catches of lionfish with a combination of spearing and hand netting has been found to concur with visual estimates of lionfish size distribution (S. Green, pers. comm.). Another source of uncertainty is the Goodyear compensation ratio (*CR*), but sensitivity analysis suggests only a minor change in SPR with a 10% increase in *CR* (Table 3).

## Discussion

Model results suggested that a high level of sustained removal would be required to reduce lionfish population sizes below the SPR threshold of recruitment overfishing. Scaling the annual exploitation rate to a lionfish per hectare removal figure based upon published data on lionfish density [7], [15], suggests a yearly removal of 157–293 lionfish per hectare would be required to cause recruitment overfishing for a population based on *M* and *CR* values of 0.5 and 15. Thus, the control of lionfish populations through targeted removal efforts will be costly, and eradication through removal efforts is highly unlikely. Intensive removals are probably only feasible at relatively small spatial scales where very high exploitation rates (i.e., >50%) can be obtained [14]. Resource managers may be able to control the invasion in a way that limits the impact of lionfish on prey species and protects ecosystem functionality, thereby protecting biodiversity and fisheries at local scales. However, before any removal program is implemented, measurable goals and target exploitation rates should be clearly defined, and pilot studies should be conducted to determine if the desired results are attainable.

Local and large-scale methods of exploiting lionfish currently exist, but need further development. On a local scale, lionfish removal events in the United States and various countries of the Caribbean have been highly successful at involving the public and generating awareness, but estimating the exploitation rate from these events is needed to measure efficacy. On a large-scale, the creation of a fishery with a high exploitation rate may produce sustainable and measurable results, but the infrastructure and demand for such a fishery does not currently exist. However, the efficacy of fishery removals would be dependent on the size at vulnerability. Furthermore, such a lionfish fishery would be limited to shallow water (<30 m) spearfishing and handnetting as lionfish have a low vulnerability to capture by hook and line [7]. This gear and depth limitation provides potential refugia from fishing, potentially making removal efforts less effective. Lionfish are being captured regularly as bycatch in reef fish trap fisheries [7], but feasibility of a lionfish specific trap capable of removing high densities of lionfish without high bycatch of native species is questionable.

This study revealed key knowledge gaps that should guide future data collection. Changes in asymptotic length (*L_∞_*) and length at 50% vulnerability to harvest (*L_vul_*) caused the greatest change in SPR. We obtained data for *K* and *L_∞_* from North Carolina, but growth parameters for lionfish could differ at more southern latitudes, and thus, more age and growth information is needed throughout the South Atlantic and broader Caribbean region. No data exist for *L_vul_*, and our values were based on a logical framework for fishes of this size, as well as personal experiences in capturing lionfish. Tagging studies should be conducted to evaluate the vulnerability of lionfish to various fishing and collection efforts, and it is likely that vulnerability will differ by region (e.g. North Carolina versus The Bahamas) as well as habitat type (e.g. mangroves versus reef versus artificial structure). Our model clearly showed that removal efforts should focus on methods to collect small lionfish, which is in agreement with other models [14].

Our model results were also sensitive to changes in natural mortality (*M*) and the Goodyear compensation ratio. No data exist on lionfish natural mortality; therefore, uncertainty surrounding the *M* parameter estimate is high. This study followed past examples [14] in using the general literature on *M* to choose a value for the model. Gathering data on *M* is a clear need and research priority. Estimates of *M* could be obtained with tagging studies or from age composition data (i.e. catch curves) in areas where lionfish are fully established but removal efforts have not occurred (i.e. total mortality = natural mortality). Additionally, there is a need for data on the Goodyear compensation ratio (*CR*). Although this parameter did not affect the model predictions as strongly as the parameters M, L_∞_, and *L_vul_*, no lionfish *CR* data exist, introducing further uncertainty in the model results.

The model was based on a simplified view of lionfish life history, which increased simulation uncertainty. Lionfish are able to spawn almost continuously [7], [12], [19], and exploited populations may receive recruits from distant source populations due to long larval duration [7], [9], [31]. Our simulation did not include contributions of larval supply from areas outside of the local population targeted by removal efforts; therefore, population recovery could occur more rapidly. The source-sink dynamics of the lionfish invasion need to be better understood, as recruitment overfishing will not be possible if recruits come from source populations that are not fished. In the current model, this occurs when small lionfish with low vulnerability to harvest are able to spawn before capture. Additionally, this could occur with a lack of removals from large spatial areas, or if source populations exist in areas that are inaccessible to removal techniques (e.g. lionfish living at depths >100 m: M. Lesser, pers. comm.).

Colonization from distant sources has been demonstrated by the success of the lionfish invasion to date [2]. Recolonization by removed invasive species is typically rapid and likely linked to their reproductive success [32], resulting in costly long-term control programs, such as with the melaleuca tree, *Melaleuca quinquenervia* (Cavanilles) [33], and zebra mussel, *Dreissena polymorpha* (Pallas) [34]. Conducting concurrent removal programs in all invaded areas would mitigate this effect, but would require large investments and may be infeasible due to the expansive and highly connected invaded range.

Because of the difficulty of substantially reducing long-term lionfish abundance through removals, the effects and goals of removal programs should be determined before proceeding. It cannot be assumed that any level of lionfish removal will be beneficial to native aquatic communities. For example, no benefits to native fishes were found after a decade of northern pike *Esox lucius* and channel catfish *Ictalurus punctatus* removals in the Colorado River and an investment of several million dollars [32]. Additionally, adult removals may cause a shift to smaller, more numerous invasive predators with the ability to consume native fish at earlier life stages [32], [35]. If this shift were to occur in lionfish on coral reefs, post-settlement mortality of native fish would likely increase, potentially driving future abundances down due to the population structuring effect of post-settlement mortality [36], [37], [38]. Studies on the biology and ecology of lionfish, interactions in the invaded community, and the efficacy of removal efforts must be conducted before committing resources to potentially ineffective removal programs.

A reasonable and measurable goal for lionfish removal efforts is to increase growth and/or abundance of native populations that have been negatively impacted by the invasion. Lionfish are known to consume and compete for food [30] and possibly shelter [6] with native fishes. Although no research on the effect of lionfish on native fish growth rates has been published, it is likely that the presence of lionfish reduces population and/or individual growth for native fishes. One study documented increased population growth in endangered California clapper rail *Rallus longirostris obsoletus* following invasive red fox *Vulpes vulpes* removal, suggesting that removal efforts can be effective at reversing a negative population growth rate trajectory [39]. Lionfish removal efforts could potentially improve abundance of native fauna by reduced predation and competition.

The complexity of the ecosystems invaded by lionfish must also be considered before enacting removal programs. Ontogenetic habitat shifts by native reef fish lead to the use of multiple essential habitat types [40], [41], [42] and make protection of ecosystem functionality an important goal. Many reef fish species use seagrass and mangrove as juvenile habitat [43], [44], [45]. Lionfish in a juvenile nursery may reduce the recruitment pool available to colonize reefs through predation or competition [6] acting in concert with lionfish predation on coral reefs [5], [30] to further stress reef fish populations. Additionally, lionfish may differentially use habitats throughout their ontogeny. Lionfish in mangrove habitat, for example, may be smaller than in reef habitat [6], suggesting mangroves may function as lionfish nurseries. If true, targeting lionfish in mangrove habitat would focus removals on the important juvenile stage, while also reducing predation stress on natives using the habitat as a nursery. This study could be used as a guide to develop targets for such control efforts before agencies invest in removal programs.
