# Correction: Evaluating the Potential Efficacy of Invasive Lionfish (*Pterois volitans*) Removals

**DOI:** 10.1371/annotation/01ecd7b0-1be0-4e2f-87c7-079d5f1a1c32

**Published:** 2011-05-31

**Authors:** Andrew B. Barbour, Michael S. Allen, Thomas K. Frazer, Krista D. Sherman

The second author's name is misspelled. The correct spelling is: Micheal S. Allen 

